# MBS Vehicle–Crossing Model for Crossing Structural Health Monitoring

**DOI:** 10.3390/s20102880

**Published:** 2020-05-19

**Authors:** Xiangming Liu, Valéri L. Markine

**Affiliations:** Department of Engineering Structures, Delft University of Technology, 2628 CN Delft, The Netherlands

**Keywords:** railway crossings, structural health monitoring, vehicle–crossing interaction, multi-body system modelling, model validation and verification, condition-stage identification

## Abstract

This paper presents the development of a multi-body system (MBS) vehicle–crossing model and its application in the structural health monitoring (SHM) of railway crossings. The vehicle and track configurations in the model were adjusted to best match the real-life situation. By using the measurement results obtained from an instrumented crossing and the simulation results from a finite element (FE) model, the MBS model was validated and verified. The results showed that the main outputs of the MBS model correlated reasonably well with those from both the measurements and the FE model. The MBS and FE models formed the basis of an integrated analysis tool, which can be applied to thoroughly study the performance of railway crossings. As part of the SHM system for railway crossings developed at Delft University of Technology, the MBS model was applied to identify the condition stage of a monitored railway crossing. The numerical results confirmed the highly degraded crossing condition. By using the measured degradation as the input in the MBS model, the primary damage sources were further verified. Through identifying the crossing condition stage and verifying the damage source, necessary and timely maintenance can be planned. These actions will help to avoid crossing failure and unexpected traffic interruptions, which will ultimately lead to sustainable railway infrastructure.

## 1. Introduction

Railway turnouts (also called switches and crossings (S&C)) are essential components of the railway infrastructure that provide the ability for the trains to transfer from one track to the other. A standard railway turnout contains three main parts:

- The switch panel that controls the train travelling directions.
- The crossing panel that provides the intersection of two tracks.
- The closure panel, which connects the other two panels.

**Figure 1:**
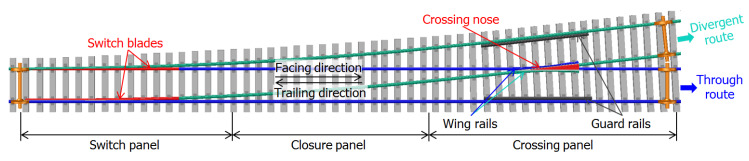
Standard left-hand railway turnout and the definition of the passing routes.

A sketch view of a standard left-hand turnout is shown in Figure 1.

When a train is passing through a turnout crossing, the wheel on the inner rail has to pass the gap between the wing rail and the crossing nose rail. The presence of this gap leads to high impact forces acting on the wing rail/crossing rail. The higher the train velocity, the higher these forces are [1,2,3]. The magnitude of the impact forces also depends on the crossing angle, namely the bigger the angle, the higher the wheel forces [3,4]. It should be noted that in contrast to the divergent route, where the maximum allowable speed of the passing trains depends on the crossing angle, there is no speed limit related to the crossing angle for the trains passing the crossing in the through route [5]. The speed limit in the through route is usually defined by the operational speed on the particular track section. Therefore, high impact forces due to the passing wheels occur on these crossings, resulting in fast degradation and short service life of the crossings. As such, the turnout crossing is one of the weakest spots in the railway network.

In recent years, the dynamic performance of railway crossings has drawn much attention and several studies on the wheel–crossing interaction and the related problems have been performed. These studies consist of both experimental [6,7,8,9,10,11,12,13,14,15,16] and numerical approaches [1,2,3,4,17,18,19,20,21,22,23,24,25,26,27,28,29,30,31,32,33,34,35,36,37]. The experimental studies can provide more realistic results than the numerical ones but are also more expensive and time-consuming. Sometimes, it is difficult to get any regular patterns from the measurement results due to the complexity of train–crossing interactions in situ [12] and the development of the structural health monitoring (SHM) system for railway crossings is still in the primary stage regarding damage detection. In such cases, numerical models are needed to help identify the crossing condition from the experimental data. For that purpose, the numerical models should be able to catch the main dynamic features of railway crossings and be suitable for performing repetitive simulations.

The numerical models for crossing performance analysis available nowadays are mainly based on the multi-body system (MBS) methods and the finite element (FE) methods. The FE models that are featured with detailed wheel–rail contact analysis are widely applied to address the local wheel–rail interaction problems. The related studies cover topics such as the development of numerical models [3,4,17], critical contact pressure [18] and stress [19] analysis, damage-related studies [20,21,22,23,24,25], methods aiming to improve the crossing performance [26], assessment of the maintenance effect [27] and damage prediction [28], etc. The primary deficiency of the FE models is the high requirement for computing resources. As it was mentioned in Ma et al. [4], one simulation of the wheel–crossing interaction may take hours or even days to finish. Therefore, the FE models are usually simplified to have only the wheel/wheelset–crossing part. Full-scaled turnout models are only applied in the static or semi-static analysis [29,30]. From this point of view, the FE models are difficult to apply for the verification of the measurement results due to the requirement of multiple trial calculations. Therefore, the MBS method that provides fast simulation is the better option. Furthermore, the MBS models are already applied in the parameter studies and design optimization of railway crossings [31,32,33,34,35,36,37], but their application combined with experimental studies are still limited, where the measurement results are mainly used for model validation.

**Figure 2:**
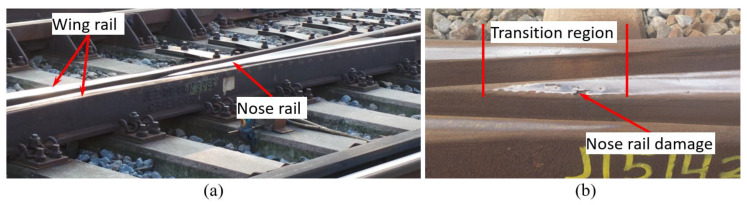
**A** 1:9 cast manganese steel crossing: (**a**) view of a wing rail and crossing nose rail, and (**b**) crossing nose rail damage in the transition region.

In the Netherlands, the most commonly used type of crossings is the cast crossing made of manganese steel with a crossing angle of 1:9 [37], as shown in Figure 2a. This type of crossing suffers greatly from severe plastic deformations and cracks, leading to spalling defects or even to sudden fracture of the crossing nose (Figure 2b). The service life of such crossings in the Netherlands has become prohibitively short, where in some cases, it is only 2–3 years [38]. To better understand the sources of their poor performance, the condition monitoring system based on instrumentation devices has been developed and implemented in this type of crossing, as described in Markine et al. [12]. The experimental data provided instant performance information about the monitored crossing, and together with the proposed condition indicators, they were used to assess the crossing condition and to describe the degradation process of the crossing. The indicators and related condition stages were determined experimentally. To verify them, as well as to extend the SHM system to other operational conditions (e.g., different types of crossings), a numerical model is necessary.

Therefore, the goal of this study was to develop an MBS model for vehicle–crossing interaction that is suitable for SHM. By using this model, both the crossing conditions and the condition indicators can be verified.

To verify the MBS model, the FE wheel–crossing model developed and validated in Ma et al. [4] was used in this study. The FE model accounted for the plastic deformation and hardening of the material on a local scale, such as the stress and strain resulting from the wheel–rail interface. The procedure of the model verification is described in this paper. After that, the use of the MBS model in the SHM system is described and demonstrated. The outline of this study is given below.

In Section 2, the modeled real-life situation, the development of the MBS model, and the input data that was adjusted according to the analyzed railway system are described. The MBS model validation using the field measurement results and the verification using the previously developed FE model are given in Section 3. Using the developed MBS model application, the identified crossing condition stage in the field monitoring is further verified, as presented in Section 4. In Section 5, the main conclusions are provided. 

## 2. MBS Model of Vehicle–Crossing Interaction

As mentioned in the previous section, one of the purposes of the MBS model was to verify the previously proposed indicators. Therefore, in this section, the monitored crossing and the obtained measurement data are presented first, followed by detailed information about the MBS model.

### 2.1. Monitored Crossings

**Figure 3:**
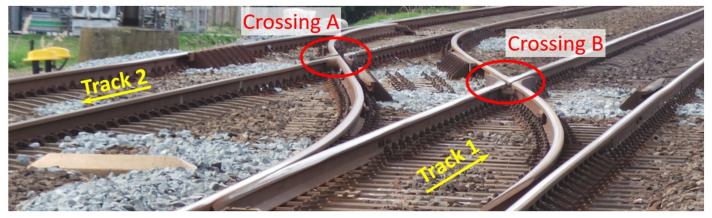
Typical crossover in the Dutch railway network.

In the railway track system, a crossover is a pair of turnouts that connects two parallel tracks and allows a train to pass over from one track to the other (Figure 3). Such a crossover is usually a part of a double crossover to allow all the trains to run on one track and gain more maintenance time for the other track. There are plenty of these crossovers on the Dutch railway network, and under normal operating conditions, the trains pass the crossings only in the through (facing or trailing) route (Figure 1). In the Dutch railway network, the presence of bridges, culverts, and level crossings limits the available space for the layout of the double crossovers. Therefore, the turnouts with the smallest crossing angle of 1:9, which requires a relatively short layout distance, are commonly used in crossovers.

**Figure 4:**
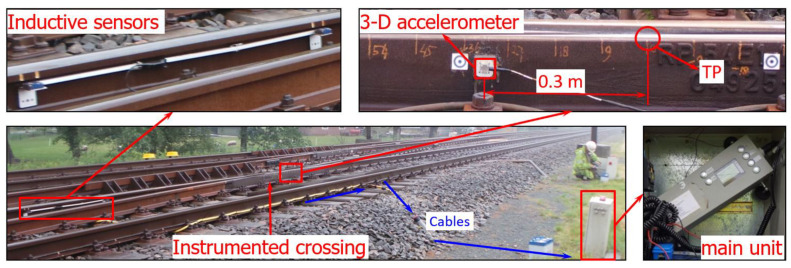
Overview of the crossing instrumentation. TP: Theoretical point.

To obtain insight regarding the crossing performance in a real-life situation and to provide guidance for the validation of the numerical models, a 1:9 cast manganese crossing from a double crossover in the Dutch railway was instrumented and monitored. The operational speed in the through route of the crossing is 140 km/h, which is much higher than the speed limit of 40 km/h in the divergent route [5]. The crossing instrumentation is based on the performance analysis device called ESAH-M, which has been introduced and actively used in previous studies [3,4,8,35]. An overview of the crossing instrumentation used in situ is shown in Figure 4.

As seen in Figure 4, the main components of this device are a 3-D accelerometer attached to the side of the crossing (0.3 m away from the theoretical point (TP)), a pair of inductive sensors attached in the closure panel, and the main unit installed near the track. The inductive sensors are used for train detection and train velocity calculations. All sensors are connected to the main unit for measurement control and data recording. The measurement range of the acceleration sensor is 500 g (≈5000 m/s^2^) and the sampling frequency is 10 kHz.

**Figure 5:**
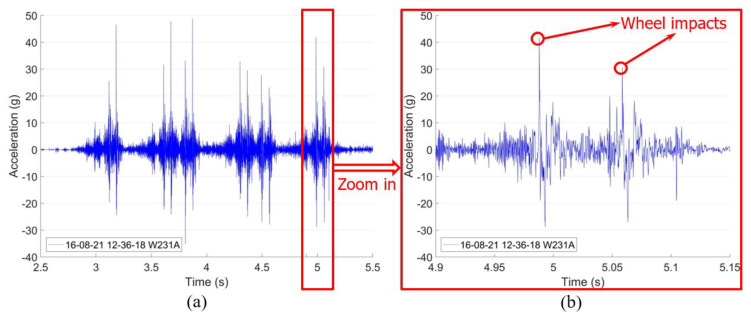
Measured crossing dynamic responses using the instrumentation: (**a**) vertical acceleration response of one train (12 wheelsets) and (**b**) examples used for wheel impacts extraction.

When a train passes through the crossing, the wheel–rail interaction is directly reflected in the vertical acceleration responses (Figure 5a). From these results, the impact due to each passing wheel can be extracted (Figure 5b). The statistical results of the impacts due to multiple passing wheels is considered to be a critical indicator for the crossing condition assessment [8].

**Figure 6:**
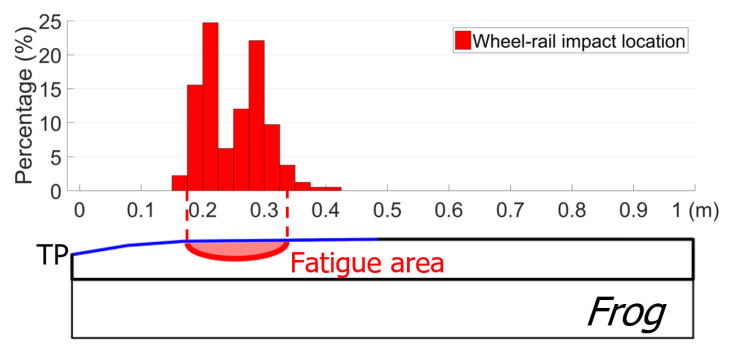
Example for the fatigue area detection.

Besides the wheel impacts, the impact location of each passing wheel could also be obtained, and the area where most of the wheel impacts were located was defined as the fatigue area, as shown in Figure 6. Practically, the fatigue area was simplified as the confidence interval of [µ−σ, µ+σ], where µ is the mean value of the wheel–rail impact locations, and σ is the standard deviation. Theoretically, 68% of the wheel impacts are located in this region. Although discrepancies exist, such a simplification can reflect the development of the wheel–rail contact condition. It has already been demonstrated in a previous study [8] that the fatigue area is a good representation of the crossing rail damage observed in the railway network, which makes it an important indicator for assessing a crossing’s condition. The wheel impacts and the fatigue area are further discussed later, in combination with the developed MBS model.

### 2.2. Geometrical Parameters

**Figure 7:**
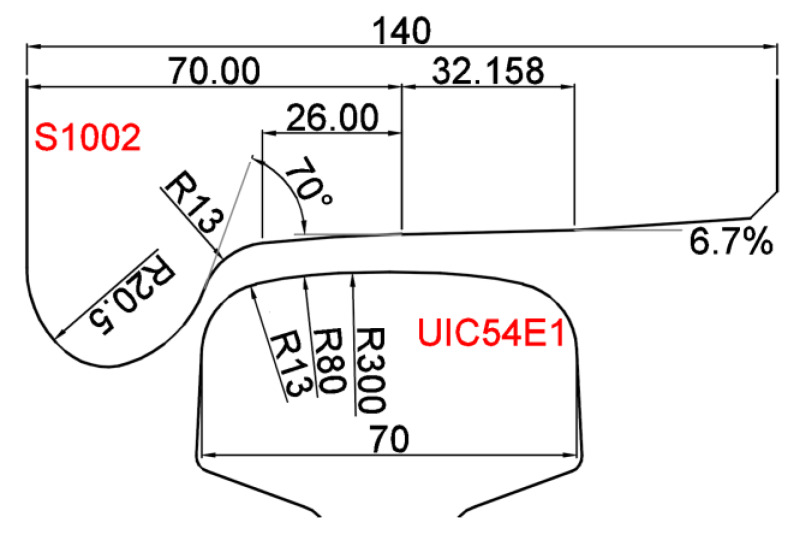
UIC54 E1 rail profile and S1002 wheel profile applied in the multi-body system (MBS) model (dimensions in mm).

In the MBS model, the vehicle model was developed based on the double-deck train VIRM [39], which is the most commonly used train type in the monitored track section. The wheel type used in the VIRM train is S1002, and the rail type used in the track is UIC54 E1. The main parameters of the S1002 wheel profile and UIC54 E1 rail profile are shown in Figure 7.

**Figure 8:**
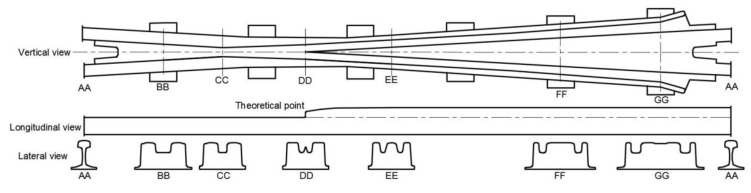
The geometry of the 1:9 cast crossing with defined critical cross-sections (drawing from Y. Ma).

The design drawing of the modeled 1:9 cast crossing is shown in Figure 8 (provided by the Dutch railway infrastructure manager ProRail). In this drawing, a group of critical cross-sections (from AA to GG) are defined to describe the crossing rail geometry. The total length of the crossing is approximately 3.7 m.

**Figure 9:**
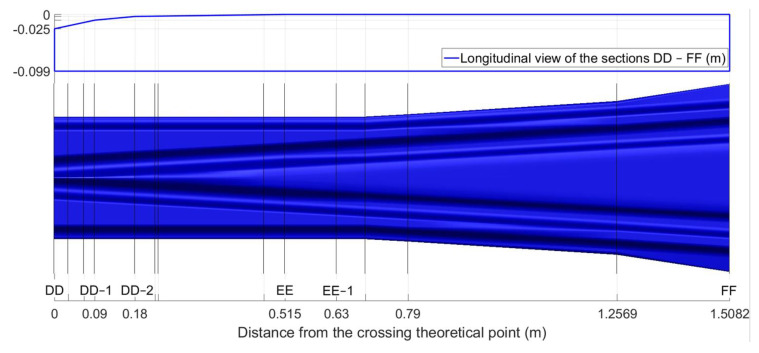
Additional cross-sections between DD and FF.

The crossing geometry is one of the critical components in the development of the MBS vehicle–crossing model. According to the design, the TP is located in the section DD, the change of the rail geometry is mainly from section CC (−0.50 m from the TP) to section FF (1.51 m from the TP). For the nose rail, the geometry is gradually developed from an arc (r = 2 mm) in the TP to the UIC54 E1 profile in the section of 0.63 m from the TP (between sections EE and FF). Some additional sections are added to precisely define the crossing geometry, which can help to control the curvature of the arcs in the rail profiles and the height of the nose rail. The additional control sections between DD and FF are shown in Figure 9.

The height of the nose rail is defined by four control sections, namely section DD, two sections every 0.09 m (DD–1 and DD–2, Figure 9), and section EE that is 0.515 m from DD. Similarly, the profile of the nose rail is defined by five control sections from the TP (DD) to the section EE–1 with the UIC54 E1 profile (0.63 m from the TP). Together with some auxiliary sections, the crossing geometry is defined by 23 control sections, including AA, and the profiles between two control sections are automatically interpolated using the third-order spline curve. 

### 2.3. Vehicle–Crossing Model

**Figure 10:**
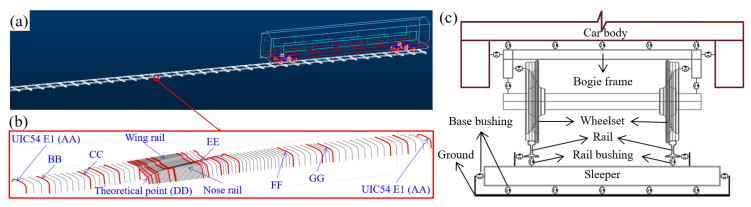
MBS model: (**a**) vehicle–track model, (**b**) flexible connections in the model, and (**c**) input crossing profiles (the control sections are marked in red).

The model for the analysis of the vehicle–track interaction developed using the MBS method (implemented in VI-Rail software is shown in Figure 10a. The track model is a straight line with the crossing panel (Figure 10b, critical sections are marked in red) situated in the middle of the track. This study concentrated on the wheel–rail interaction in the crossing panel. Therefore, the switch panel (Figure 1) was simplified to a normal track. The profiles between two adjacent sections were automatically interpolated using the third-order spline curve. The total length of the track model was 100 m, which allowed for enough preloading space (around 1 m) before the vehicle entered into the crossing panel, as well as enough space after the vehicle passed through the crossing.

The vehicle model was developed based on the VIRM train model with a total length of 27.5 m. The car body and bogie frames, as well as the wheelsets, were modeled as rigid bodies with both primary suspension and secondary suspension taken into account (Figure 10c). The wheel–rail contact model was defined as the general contact element that used the actual wheel and rail profiles as the input, which allowed for variable wheel and rail profiles and a visualized contact graphic.

In the MBS simulation, the main outputs included the wheel displacements, rail accelerations (optional), wheel–rail contact forces and contact area, etc. The computation of the wheel–rail contact force was based on the Hertz contact theory. The elastic deformation was estimated according to the undeformed penetration, which was used for the contact area calculation. With the contact force and contact area, the wheel–rail contact pressure could be obtained. More information about the methodologies can be found in VI-Rail documentation [40].

### 2.4. Model Parameters

Before the simulations, the properties of the track and the corresponding elements in the MBS models were thoroughly checked and adjusted to ensure that the MBS model and the FE model (used for the model verification) described the same real-life railway system (the monitored crossing). The vehicle/wheelset properties used in the MBS model are given in Table 1. The total axle load was calculated from the wheelset, bogie, and car body masses, which was 10 t in this model. In the FE model [4], the axle load was also 10 t, while the weights of the bogies and the car body were all integrated into the simplified half-moving wheelset.

The main properties of the rail model were Young’s modulus and density. For the rail pad and ballast, the stiffness and damping in both vertical and lateral directions were taken into account. The main track properties are given in Table 2, referring to Hiensch et al. [41].

## 3. Model Validation and Verification

In the previous study [4], the FE wheel–crossing model for the crossing performance analysis was already developed and validated. The explicit FE model can take the plastic deformation and hardening of the material on a local scale into account, which is quite helpful for a better understanding of the wheel–rail interaction. To allow for the combination of the MBS model with the FE model to thoroughly study the dynamic performance of railway crossings, it is of great importance that the MBS model is not only comparable with the measurement results but also close to the output of the FE model. Therefore, the developed vehicle–crossing MBS model was validated using the measurement results from the crossing instrumentation and verified using the simulation results from the FE model.

To better compare with the measurement and FE simulation results, the train running direction was set to the facing through route and the time step was adjusted to 0.0001 s, which was consistent with that in the FE simulation and the sampling frequency of the measurement data. The following response quantities that reflect the performance of the crossing were used to validate and verify the MBS model:

- The transition region where the wheel load transitioned from the wing rail to the nose rail, which is considered the most vulnerable region in the crossing.
- The vertical impact acceleration within the transition region.

Furthermore, some other output data from both the MBS and the FE simulations, including the vertical wheel trajectory and contact forces, were compared further to prove the compatibility of the two numerical models. All these results are presented and analyzed in the following sections.

### 3.1. Transition Region

**Figure 11:**
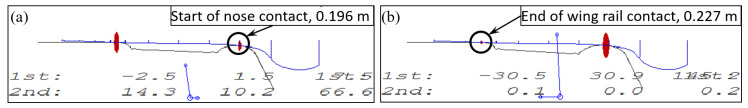
Transition region calculation in the MBS model: (**a**) start contact with the nose rail and (**b**) end contact with the wing rail.

In the MBS simulation, the transition region was where the wheel and crossing rail had two-point contact and was recognized as the interval between the start of wheel–nose rail contact and the end of the wheel–wing rail contact. The size and location of the transition region reflected the smoothness of the wheel–rail contact transition from the wing rail to the nose rail. The transition region calculated using the MBS model was 0.196–0.227 m, as shown in Figure 11. 

**Figure 12:**
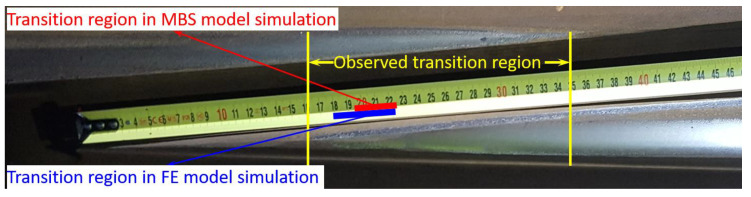
Transition regions obtained from the field observation and the numerical simulations. FE: finite element.

In real life, the transition region was obtained through inspection and recognized as the overlapping shining bands on both the wing rail and the crossing nose. For the monitored crossing, the observed transition region was around 0.16–0.35 m with a size of 0.19 m, as shown in Figure 12. It can be seen that the transition region in the MBS simulation was within the observed one but was much smaller with a size of only 0.031 m. Such a phenomenon can be explained by the ideal initial conditions (no lateral angle or displacement) of the wheels used in the simulations and the absence of the wheel or rail irregularities. Moreover, the actual crossing was not new and had a certain level of plastic deformations and wear. In reality, every wheel passed the crossing with a certain angle and lateral shift that resulted in earlier/later contact in the transition region. The fact that the simulated transition region was included in the transition region of the real crossing proved the correctness of the MBS simulation results.

The transition region in the FE model simulation [4] was 0.180–0.223 m with a size of 0.043 m, which was 30% larger than that obtained from the MBS simulation. Considering that in the MBS model, the wheels and rails were simulated as rigid bodies without taking the material deformation into account, the transition regions in both methods were close to each other, which proved the compatibility of the MBS models with the FE model.

### 3.2. Impact Acceleration and Fatigue Area

**Figure 13:**
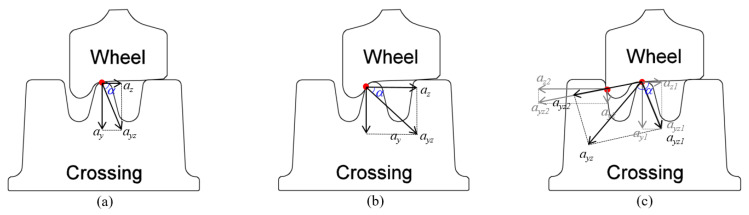
Wheel–rail contact situations: (**a**) regular contact, (**b**) irregular (positive) flange–nose rail contact, and (**c**) irregular (negative) flange–wing rail contact.

Due to the uncertainty of the wheel–rail contact situation, the measured impact accelerations of the passing wheels can vary a lot from one to another in terms of amplitude and impact angles [8], as shown in Figure 13. In extreme cases, such as the wheel flange impact on the rail (Figure 13b,c), the acceleration responses can be up to 10 times higher than that due to a normal passing wheel.

**Figure 14:**
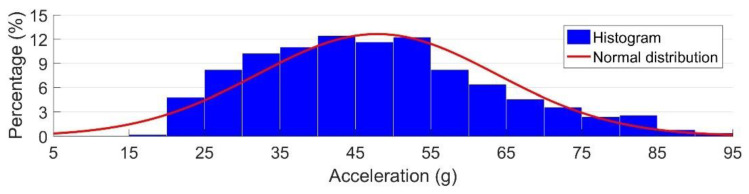
Histogram of the measured vertical accelerations used in the model validation.

The measured acceleration signals for the model validation contained more than 1000 wheels from 90 trains. In both numerical models, no track or rail irregularities were considered, which means that in the numerical simulations, the wheel (wheel-set) did not experience any additional disturbance when passing the crossing. As a result, the contact situation in these simulations was always regular (Figure 13a). Therefore in the model validation, only the measured signals with the regular contact (|*a_y_*|>|*a_z_*|) were used, which resulted in 500 selected passing wheels. The distribution of the impact accelerations due to these passing wheels is shown in Figure 14. The resulting histogram can be considered a normal distribution, with a mean value of μ = 47.15 g and a standard deviation of σ = 17.65 g.

**Figure 15:**
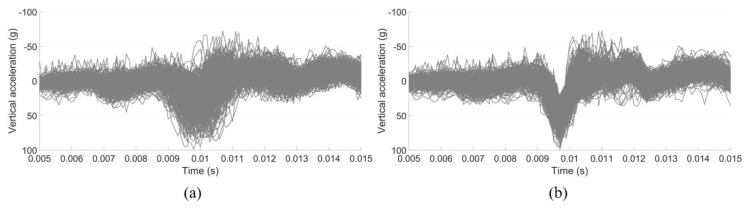
Measured acceleration responses: (**a**) original time−domain results and (**b**) modified results, with the time histories aligned horizontally with the impact point.

The time-domain representation of the selected measured acceleration responses used in Figure 14 is given in Figure 15a. For a better interpretation, the time histories were aligned horizontally with the wheel–rail impact point (Figure 15b), which were used for validation of the numerical model.

#### 3.2.1. Impact Acceleration Analysis

**Figure 16:**
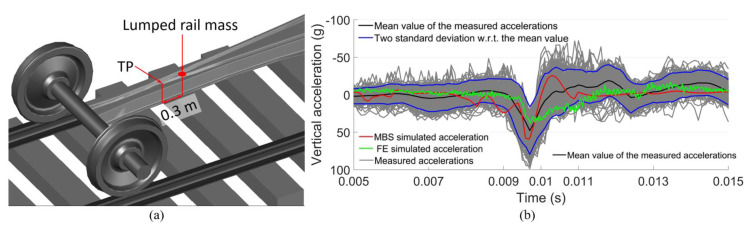
(**a**) Rail element for acceleration extraction in the MBS model. (**b**) Comparison of simulated accelerations with measured ones in time domain.

In the MBS model, the selected element used for the acceleration extraction was the rail with a lumped mass (Figure 16a) located 0.3 m from the TP, which was the same as the location of the accelerometer in the crossing instrumentation (Figure 4). The comparison of the MBS simulation results with the measured responses and the FE simulation results is shown in Figure 16b.

From Figure 16b, it can be seen that the amplitude of the MBS simulated vertical acceleration was higher than the mean value of the measured acceleration, as well as those from the FE simulation. It can also be noted that some of the measured accelerations had rebound after the impact (0.01–0.011 s). The MBS simulation also had such a rebound, while the FE simulation did not.

The discrepancy between the MBS and FE simulations were mainly due to the different assumptions in these models. In the MBS model, the wheelsets, rails, and sleepers were all modeled as rigid bodies. In this case, the elasticity and damping of the vehicle–track system were underestimated, which led to the higher amplitude of the rail acceleration. While in the FE model, the crossing rail was modeled as a solid element without a hollow inside. This means that the rail mass and stiffness were overestimated, which resulted in relatively small accelerations. Nevertheless, both simulation results were located within the interval [µ−σ, µ+σ] of the measured accelerations, meaning that although tolerable discrepancies existed, the MBS model was reasonably compatible with the field measurements, as well as with the FE model.

#### 3.2.2. Fatigue Area Analysis

**Figure 17:**
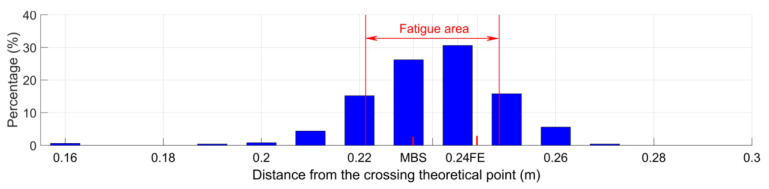
Distribution of wheel impact locations.

The distribution of the wheel impact locations for the selected measurement data is shown in Figure 17. Based on these results, the fatigue area of the crossing was calculated, which was 0.221–0.249 m from the TP. In the MBS simulation, the wheel impact was located at 0.231 m from the TP, which was very close to the center of the fatigue area, as marked in Figure 17. The fatigue area obtained from field measurement represents the degree of concentration of the wheel impacts, while the impact location in the MBS simulation was only from one wheel passage. Even so, the close results proved the correctness of the MBS model.

By comparison, the impact location in the FE simulation was 0.244 m, which was within the fatigue area as well. The close impact locations obtained from the MBS and the FE simulations further proved the compatibility of these two models.

It must be noted that the wheel impacts and the fatigue area were calculated based on the selected wheels, which all involved in regular wheel–rail contacts, and the deviation was quite limited. Therefore, the resulting wheel impacts and fatigue area could not fully represent the real-life situation, and therefore should not be used to assess the crossing condition.

### 3.3. Other Responses

In the numerical simulations, the wheel trajectory was related to the global responses of the models and characterized the correctness of the geometry representation in the models. On the other hand, the wheel–rail contact forces were related to the local properties and reflected the accuracy of the modeling of the wheel–rail contact. To further verify the compatibility of these two models, the vertical wheel trajectory, as well as the wheel–rail contact forces, were compared. The results and analysis are presented below. For the MBS simulation, the results from the first wheelset of the vehicle were applied.

#### 3.3.1. Wheel Vertical Trajectory

**Figure 18:**
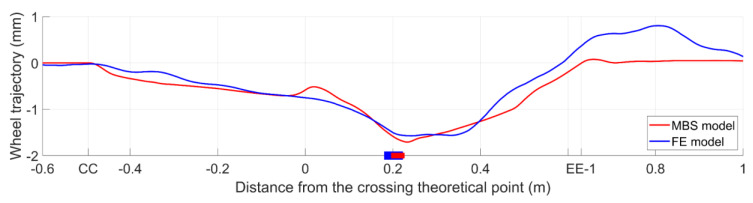
Wheel vertical trajectory comparison.

The vertical wheel trajectory is the vertical displacement of the wheel relative to the rail. The change of the trajectory reflects the smoothness of the wheel passing through the crossing [35]. The vertical trajectories of the wheel in the MBS and FE simulations are shown in Figure 18. To provide a better comparison, the initial points of all the simulations were shifted to zero.

It can be seen from Figure 18 that despite some slight differences near the TP and after the transition region, the trajectories of both simulations were very close to each other. The maximum displacement in the MBS simulation was 1.71 mm at 0.231 m from the TP, while in the FE simulation, it was 1.57 mm at 0.242 m from the TP. The difference between these two models was only 9%. It can be seen that the maximum values in both simulations occurred shortly after the transition of the wheel load, which was consistent with the impact acceleration.

In the MBS simulation, the trajectory showed several abrupt changes where the rail geometries were variated (e.g., in sections of CC, DD (TP), and EE–1). Such a development was in accord with the rigid body assumption in the MBS model. By comparison, the trajectory was more gently developed in the FE simulation, except for the slight rebound after section EE–1. Such a phenomenon can be explained by the fact that in the FE model, the wheel and rails were modeled as solid elements that allowed the material to deform. The material deformation due to the wheel–rail contact reduced the influence of small rail geometry variations. After the wheel passed the variated region (sections CC – EE–1), the released wheel load led to the resilience of the rail and resulted in the slight rebound.

#### 3.3.2. Vertical Contact Forces

**Figure 19:**
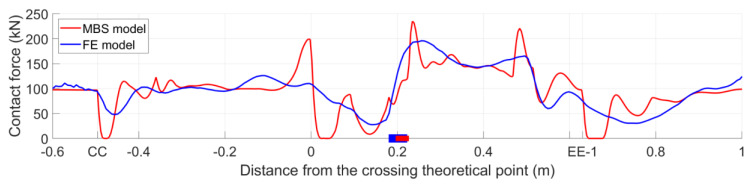
Comparison of the vertical wheel−rail contact forces.

Figure 19 shows the vertical wheel–rail contact forces obtained from the MBS and FE models. Similar to the wheel trajectory comparison, the contact forces of both models were close to each other. In the MBS simulation, loss of wheel–rail contact occurred near the sections of CC, DD, and EE–1, which were consistent with the locations where the wheel slightly rebounded (Figure 19). By comparison, the wheel–rail contact forces in the FE model developed smoother than those in the MBS models with less fluctuation.

The decrease in the contact forces of both models near section CC (Figure 19) indicated the beginning of the wing rail. At this point, the wheel–rail contact point on the wheel shifted farther from the wheel flange. In the MBS model, the sudden increase of the contact force near the TP reflected the effect of geometry change of the wing rail. It must be noted that the first peak values (after passing through the TP) of both models occurred after the respective transition regions. In the MBS simulation, the pick value was 235 kN, which was located at 0.235 m from the TP. Meanwhile, in the FE model, it was 196 kN at 0.256 m. The second peak values were 221 kN at 0.484 m in the MBS model and 165 kN at 0.496 m in the FE model.

It can be concluded that the contact forces obtained from the MBS model were comparable to those from the FE model. Some saltation in the MBS simulation was caused by modeling the wheel and rail elements as rigid bodies without considering the flexibilities of them. The slight hysteresis of the contact force calculation in the FE model was due to the effect of material deformation. From this point of view, the FE simulation was closer to the real situation. Even so, as a much more efficient alternative, the MBS model can also provide acceptable results.

The comparable results of the MBS model simulation with the FE model simulation further confirmed that both models described the same real-life system. For the same simulation presented in this section, the calculation time of the FE model was a few days, while that of the MBS model was only a few minutes. Therefore, the MBS model could be better applied in repetitive simulations, such as rail geometry optimization and track irregularity analysis. For the dynamic performance analysis of railway crossings, this MBS model can be applied for the preliminary simulations to find out the critical situations. The obtained critical situations can then be used as the inputs into the FE model for detailed wheel–rail contact analysis. The combined MBS–FE methods form an integrated tool that can be applied to thoroughly study the dynamic performance of railway crossings. 

In this section, the developed MBS model was validated and verified using both the measured results and the FE simulation results. Although tolerable discrepancies existed, the MBS model was reasonably compatible with field measurement and the FE model. It can be concluded that the MBS model can identify the main features of the wheel–rail impact at a crossing and can be used to analyze the crossing performance.

## 4. Application in Crossing Condition Monitoring

In the crossing condition monitoring tool developed in [13], the condition assessment was made based on the changes in the dynamic performance indicators during the monitored period. In some cases, when the monitoring has to be performed on an already operating (not newly installed) crossing, its condition stage at that moment is difficult to determine, especially for a new type of crossing for which no monitored history is available. With the help of the MBS model developed in this study, the condition stage can be determined by comparing the measurement results with the simulation results that are based on the new (designed) crossing condition.

In a case where the monitored crossing is identified to be in a degraded condition, the damage sources will need to be inspected. The inspected crossing damage can be then used as the input into the MBS model to simulate the crossing performance in the degraded condition. By comparing the simulation results with the measured ones, the damage sources of the crossing degradation can be verified. By knowing the crossing damage sources, proper maintenance actions can be implemented in a timely manner to avoid fatal defects and unexpected track disruptions.

In this section, the above-mentioned applications (identify condition stages and verify damage sources) are demonstrated in a monitored 1:9 trailing crossing, as presented below.

### 4.1. Condition Stage Identification

The studied 1:9 trailing crossing was located in the same double crossover as the facing crossing used for model validation in Section 3. Similarly, the trains were mainly passing the crossing mainly on the through route with velocities up to 140 km/h. In contrast to the crossing analyzed in Section 3, this crossing was passed in the trailing direction. Nevertheless, the same MBS model presented in Section 2 was used here to assess the crossing performance. The in situ performance of the crossing was obtained using the instrumentation (Figure 4). By comparing the measurement results with the simulation results of the crossing in the designed condition, the actual condition stage of the crossing was identified.

#### 4.1.1. Measurement Results and Analysis

**Figure 20:**
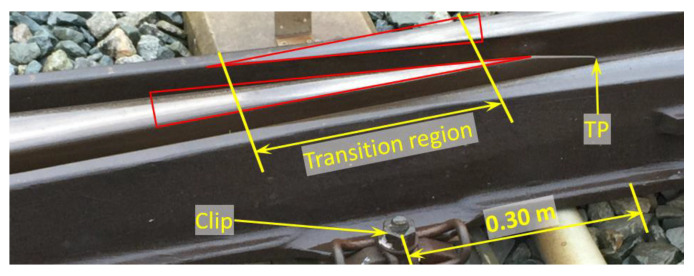
Transition region inspection of the monitored 1:9 trailing crossing.

To process the measured data, the transition region in the crossing was inspected, as shown in Figure 20. The transition region was recognized as the region with overlapping shining bands. Using the track dimensions (the sleeper width was 0.20 m, and the clip was located at 0.30 m from the TP), the transition region of this crossing was located between 0.15–0.40 m from the TP.

**Figure 21:**
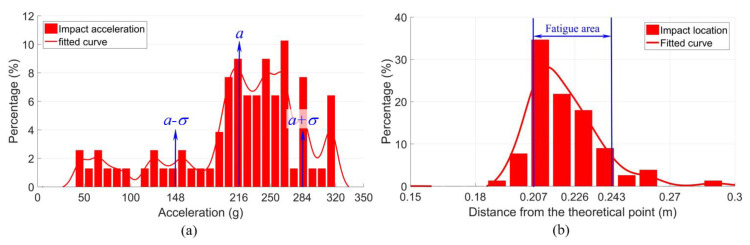
Measured dynamic responses: (**a**) wheel impact distribution and (**b**) impact location distribution.

The measurement data used for the crossing performance analysis consisted of the multiple wheel passages from one monitoring day. To be consistent with the numerical simulation, only the results from the VIRM trains with velocities of around 140 km/h, as used in the model, were selected, which resulted in a sample size of 78 passing wheels. The magnitude and location of the impacts due to these wheels were analyzed and the results are presented in Figure 21. 

Figure 21a shows the magnitude distribution of the measured impact acceleration responses. The mean value was 216 g and the standard deviation was 68 g. The impact location distribution is shown in Figure 21b, from which it can be seen that the majority of the wheel impacts (the fatigue area) was located at a distance 0.207–0.243 m from the TP, resulting in a size of the fatigue area of 0.036 m.

The wheel-impact-based results were the most representative ones that reflected the condition of the crossing. In the next section, these results are compared with the simulation results of the crossing in the designed condition to identify the actual condition stage of the monitored crossing.

#### 4.1.2. Numerical Simulation and Condition Stage Identification

The MBS vehicle–crossing model used here to analyze the crossing performance in the designed condition was the same as the one presented in Section 2. The only difference was that the vehicle was now moving in the trailing direction, meaning that the wheel load on the crossing panel was transferred from the crossing nose to the wing rail. Using the designed (not worn) crossing shape and the other model parameters given in Section 2, the dynamic performance of the 1:9 trailing crossing was analyzed. 

**Figure 22:**
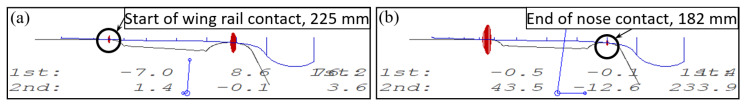
Transition region calculation of a 1:9 trailing crossing in the designed condition: (**a**) start of the contact with the wing rail and (**b**) end of the contact with the nose rail.

The determination of the transition region using the simulation results is demonstrated in Figure 22. In contrast with the facing crossing, the wheel load in the trailing crossing moved from the crossing nose to the wing rail. Therefore, the transition region started from the wheel–wing rail contact (Figure 22a) and ended with the loss of wheel–crossing nose contact (Figure 22b). The determined transition region was 0.182–0.225 m from the TP. This region was located within the one obtained during the field inspection (0.15–0.40 m), as shown in Figure 20. Thus, this confirmed that the MBS model developed for the trains passing in the facing direction was also valid for the trailing crossing analysis.

**Figure 23:**
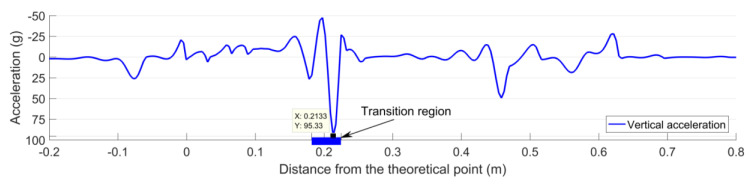
Rail vertical acceleration responses of a 1:9 trailing crossing in designed condition.

Figure 23 shows the vertical acceleration responses of the crossing rail due to the first passing wheel. It can be seen that the maximum acceleration (due to the wheel impact) was 95 g. This value was much lower than the mean value of the measured impact acceleration (216 g) shown in Figure 21a. Based on the significant difference (increase) between the measured and the simulated crossing accelerations (in the designed condition), it can be concluded that the monitored crossing was in a highly degraded condition. This conclusion was in agreement with the experimental results of a 1:15 crossing presented in [13], wherein the significant increase (68%) in the observed measured acceleration was correlated with the visible damage of the crossing rail.

The fatigue area in the designed condition could not be determined from the numerical simulation. Yet, the wheel impact location could be obtained, which was 0.213 m from the TP (Figure 23). Similar to the transition region, the impact location was within the measured fatigue area (0.207–0.243 m, Figure 21b).

It can be seen that in the degraded condition, the main change was the increased wheel impact acceleration, while the change in the impact location was rather limited. In the next step, the damage sources of this crossing were detected and verified, as presented in the next section.

### 4.2. Damage Source Detection and Verification

A typical SHM consists of five levels of activities, namely detection, localization, assessment, prediction, and remediation [42]. In the previous section, the highly degraded condition of the monitored crossing was identified, which corresponded to the first step of an SHM (determine the presence of structural damage). Then, the second step was to localize the damage to guide crossing maintenance. 

#### 4.2.1. Degraded Crossing Geometry

**Figure 24:**
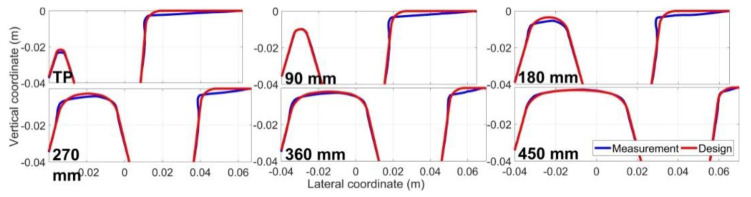
Measured crossing profiles in critical sections compared with the designed profiles.

For a regularly degraded railway crossing, one of the typical damage sources is rail wear and deformation. For the monitored crossing, the rail profiles in the critical sections were measured and compared with the designed profiles, as shown in Figure 24.

It can be seen from Figure 24 that the crossing rail was worn and deformed. The most severe material damage on both the wing rail and the nose rail occurred in the section of 0.18–0.27 m, which was consistent with the distribution of the wheel impact locations (0.207–0.243 m, Figure 21b). It can also be seen that the wear and deformation of the wing rail continued into the section of 0.00–0.18 m, meaning that the rail degradation extended out of the transition region. From this point of view, the crossing had been operating under a degraded condition for a significant period.

#### 4.2.2. Numerical Verification

The geometry measurement results presented in the previous section indicated the worn and deformed condition of the crossing and wing rails. To verify the effect of this damage on the crossing performance, the measured rail profiles were implemented in the MBS model and the numerical simulations were performed again. 

**Figure 25:**
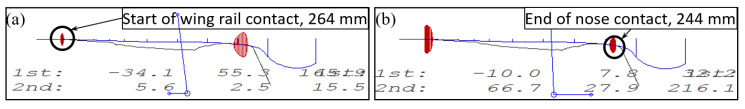
Transition region calculation of a 1:9 trailing crossing using the measured rail geometry: (**a**) start of the contact with the wing rail and (**b**) end of the contact with the nose rail.

The calculated transition region was 0.244–0.264 m, as shown in Figure 25. It can be seen that due to the severe wear and deformation of the wing rail, the initial wheel–wing rail contact (Figure 25a) occurred earlier than that in the designed condition (0.225 m, Figure 22a). Furthermore, the size of the transition region was reduced to only 0.020 m (compared with 0.043 m in the designed condition). The damaged rail geometry resulted in the transition region being shifted further away from the TP and there was a sharper transit of the wheel load from the crossing nose to the wing rail. The narrowed transition region with a shift farther from the TP can indicate a degraded crossing rail geometry. Such a changing pattern is in agreement with the development of the fatigue area that was observed in the previous study of a 1:15 facing crossing in [13].

**Figure 26:**
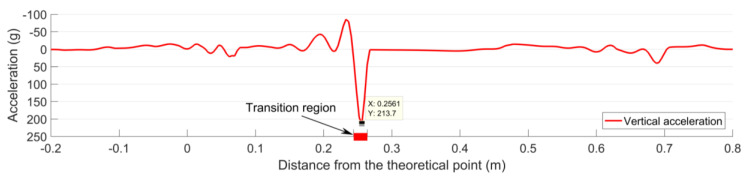
Simulation results of the 1:9 trailing crossing with rail wear and deformation taken into account.

The simulation results of the crossing acceleration due to the first passing wheel is shown in Figure 26. Compared with the designed condition (Figure 23), the crossing acceleration in the degraded condition was increased from 95 g to 214 g, and such a result was quite close to the mean value of the measured results (216 g, Figure 21a). These results indicated that the degraded rail geometry was the main cause of the increased accelerations. 

It should be noted that the simulated wheel impact was located at 0.256 m from the TP, which was not consistent with the measured fatigue area (0.207–0.243 m, Figure 21b). Furthermore, this location was 0.043 m farther than the wheel impact in the designed condition (0.213 m, Figure 23). Such a result indicated that besides the degraded crossing rail geometry, there might also be some other degraded elements in the monitored crossing (e.g., uneven ballast settlement) that need to be further investigated in combination with displacement measurement results.

**Figure 27:**
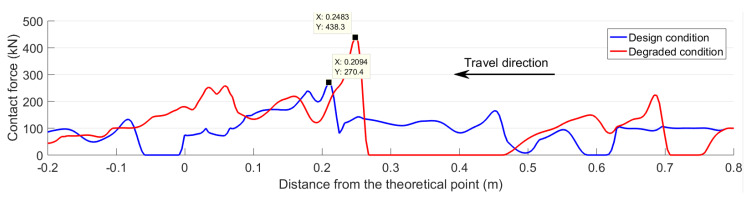
Wheel−rail contact forces in different rail conditions.

To analyze the developments in the wheel–rail interaction, the contact forces in both the designed and degraded conditions were also compared. The results are presented in Figure 27. Besides the dramatically increased impact force (438 kN in the degraded condition vs. 270 kN in the designed condition), the rail damage resulted in the loss of wheel–rail contact in the 0.27–0.46 m region. Such results show the influence of rail wear and deformation on the wheel’s behavior. Due to the interaction with the other wheel from the same wheelset, as well as the influence of the other wheelset within the same bogie, the wheel that was running through the crossing could not fully follow the damaged rail profile. Consequently, when the wheel resumed contact with the rail, the contact force was dramatically increased and the wheel load was sharply shifted from the crossing nose to the wing rail. Such a sharp transition of the wheel load led to a higher impact on the crossing.

Based on the results and analysis in this section, it can be seen that the developed MBS model was successfully applied to help identify the condition stage of a crossing and to verify the damage sources in combination with field inspection. Furthermore, it can be concluded that the monitored 1:9 trailing crossing was in a highly degraded condition. The high wheel–rail impacts were mainly correlated with the worn and deformed rail geometry. Repair welding and grinding in this crossing are urgently required to avoid further damage (e.g., cracks, spalling, etc.).

The application of the MBS model in the condition monitoring of the 1:9 trailing crossing further confirmed that the condition indicators (proposed based on 1:15 facing crossing in the previous study [13]) are applicable for different types of crossings (e.g., angle, traffic direction, etc.), which provides a better opportunity for the promotion of the condition monitoring system.

The deformed crossing geometry in the studied crossing was the dominant factor causing the degradation, though there is still likely to be some other damage that was not detected, e.g., ballast settlement, track misalignment, etc. The developed MBS model proved to be sufficient for the crossing condition stage identification and damage source verification, yet a track inspection was still required. A better way to master the crossing condition is to combine the MBS model with the condition monitoring. With sufficiently detected damage sources, proper and timely maintenance actions can be planned, which will help improve the crossing performance and ultimately lead to sustainable railway crossings.

## 5. Conclusions

In this study, an MBS model for the crossing performance analysis was developed. The model was validated and verified using the field measurement results and the FE simulation results. With the assistance of this MBS vehicle–crossing model, the condition stage of a monitored crossing was identified, and the source of the crossing damage is verified. Based on the results and analysis, the following conclusions can be drawn.

The MBS model was validated and verified using the field measurement and FE simulation results. The comparable results of the transition region, wheel impact acceleration, and impact location proved the validity of the MBS model for the wheel–crossing dynamic analysis. The comparison of the simulation results from the MBS model and the FE model correlated very well. It was verified that the MBS model could identify the main features of the crossing’s dynamic behavior. The differences between these two models could be explained by the different simplifications in each model, such as the simplified beam element of the rail (instead of solid element) in the MBS model and using half a wheelset (instead of a whole wheelset or bogie) in the FE model.

The MBS model displayed fast simulations, which is suitable for analysis that requires repetitive simulations. Therefore, it can be applied in the preliminary analysis (e.g., parametric study, rail geometry optimization, and track irregularity analysis) seeking critical operating conditions. The FE model can then be used to perform detailed wheel–rail contact analysis based on the critical conditions obtained from the MBS simulations. 

Using the developed MBS vehicle–crossing model, the degraded condition stage of a monitored 1:9 trailing crossing was successfully identified. The rail wear and deformation were further verified as the primary damage sources. The simulation results proved that the rail damage of the crossing was already at a severe level, showing a highly deteriorated crossing performance. With the assistance of the MBS model, the procedure for the crossing condition assessment can be dramatically simplified.

Combined with the simulation results, the measurement results regarding the degraded 1:9 trailing crossing also proved the applicability of the condition indicators for different crossing types (e.g., crossing angle, travel directions, etc.), which provides a sound basis for promoting the indicators in the condition monitoring of railway crossings.

The MBS model is a necessary supplement to the SHM system for railway crossings. Through identifying the crossing condition stage, necessary crossing maintenance actions can be better planned and implemented in a timely manner, which can help avoid fatal crossing damage and unexpected traffic interruptions. With sufficient condition information of railway crossings, the crossing maintenance strategy can be ultimately improved from “failure reactive” to “failure proactive” and lead to sustainable railway crossings with better performances.

## Figures and Tables

**Table 1:** Track parameters.

Item		Value
Wheel	Profile	S1002
	Radius (m)	0.46
Wheelset	Mass (kg)	1100
Bogie	Mass (kg)	3800
Car body	Mass (kg)	68000

**Table 2:** Track properties of the MBS model.

Track Component		Value
Rail	Young’s modulus (GPa)	210
	Mass density (kg/m^3^)	7900
Rail pad	Vertical stiffness (MN/m)	1300
	Vertical Damping (kN·s/m)	45
	Lateral stiffness (MN/m)	280
	Lateral Damping (kN·s/m)	58
Ballast	Vertical stiffness (MN/m)	45
	Vertical Damping (kN·s/m)	32
	Lateral stiffness (MN/m)	45
	Lateral Damping (kN·s/m)	32
